# DNA-Sequence Variation Among *Schistosoma mekongi* Populations and Related Taxa; Phylogeography and the Current Distribution of Asian Schistosomiasis

**DOI:** 10.1371/journal.pntd.0000200

**Published:** 2008-03-19

**Authors:** Stephen W. Attwood, Farrah A. Fatih, E. Suchart Upatham

**Affiliations:** 1 State Key Laboratory of Biotherapy, West China Hospital, West China Medical School, Sichuan University, Chengdu, People's Republic of China; 2 Department of Zoology, The Natural History Museum, London, United Kingdom; 3 Department of Biology, Faculty of Science, Mahidol University, Bangkok, Thailand; 4 Department of Medical Science, Faculty of Science, Burapha University, Bangsaen, Chonburi, Thailand; James Cook University, Australia

## Abstract

**Background:**

Schistosomiasis in humans along the lower Mekong River has proven a persistent public health problem in the region. The causative agent is the parasite *Schistosoma mekongi* (Trematoda: Digenea). A new transmission focus is reported, as well as the first study of genetic variation among *S. mekongi* populations. The aim is to confirm the identity of the species involved at each known focus of Mekong schistosomiasis transmission, to examine historical relationships among the populations and related taxa, and to provide data for use (*a priori*) in further studies of the origins, radiation, and future dispersal capabilities of *S. mekongi*.

**Methodology/Principal Findings:**

DNA sequence data are presented for four populations of *S. mekongi* from Cambodia and southern Laos, three of which were distinguishable at the COI (*cox1*) and 12S (*rrnS*) mitochondrial loci sampled. A phylogeny was estimated for these populations and the other members of the *Schistosoma sinensium* group. The study provides new DNA sequence data for three new populations and one new locus/population combination. A Bayesian approach is used to estimate divergence dates for events within the *S. sinensium* group and among the *S. mekongi* populations.

**Conclusions/Significance:**

The date estimates are consistent with phylogeographical hypotheses describing a Pliocene radiation of the *S. sinensium* group and a mid-Pleistocene invasion of Southeast Asia by *S. mekongi*. The date estimates also provide Bayesian priors for future work on the evolution of *S. mekongi*. The public health implications of *S. mekongi* transmission outside the lower Mekong River are also discussed.

## Author Summary

Schistosomiasis is a disease caused by parasitic worms of the genus *Schistosoma*. In the lower Mekong river, schistosomiasis in humans is called Mekong schistosomiasis and is caused by *Schistosoma mekongi*. In the past, Mekong schistosomiasis was known only from the lower Mekong river. Here DNA-sequence variation is used to study the relationships and history of populations of *S. mekongi*. Populations from other rivers are compared and shown to be *S. mekongi*, thus confirming that this species is not restricted to only a small section of one river. The dates of divergence among populations are also estimated. Prior to this study it was assumed that *S. mekongi* originated in Yunnan, China, migrated southwards across Laos and into Cambodia, later becoming extinct in Laos (due to conditions unsuitable for transmission). In contrast, the dates estimated here indicate that *S. mekongi* entered Cambodia from Vietnam, 2.5–1 Ma. The pattern of genetic variation fits better with a more recent, and ongoing, northwards migration from Cambodia into Laos. The implications are that Mekong schistosomiasis is more widespread than once thought and that the human population at risk is up to 10 times greater than originally estimated. There is also an increased possibility of the spread of Mekong schistosomiasis across Laos.

## Introduction

**Figure 1:**
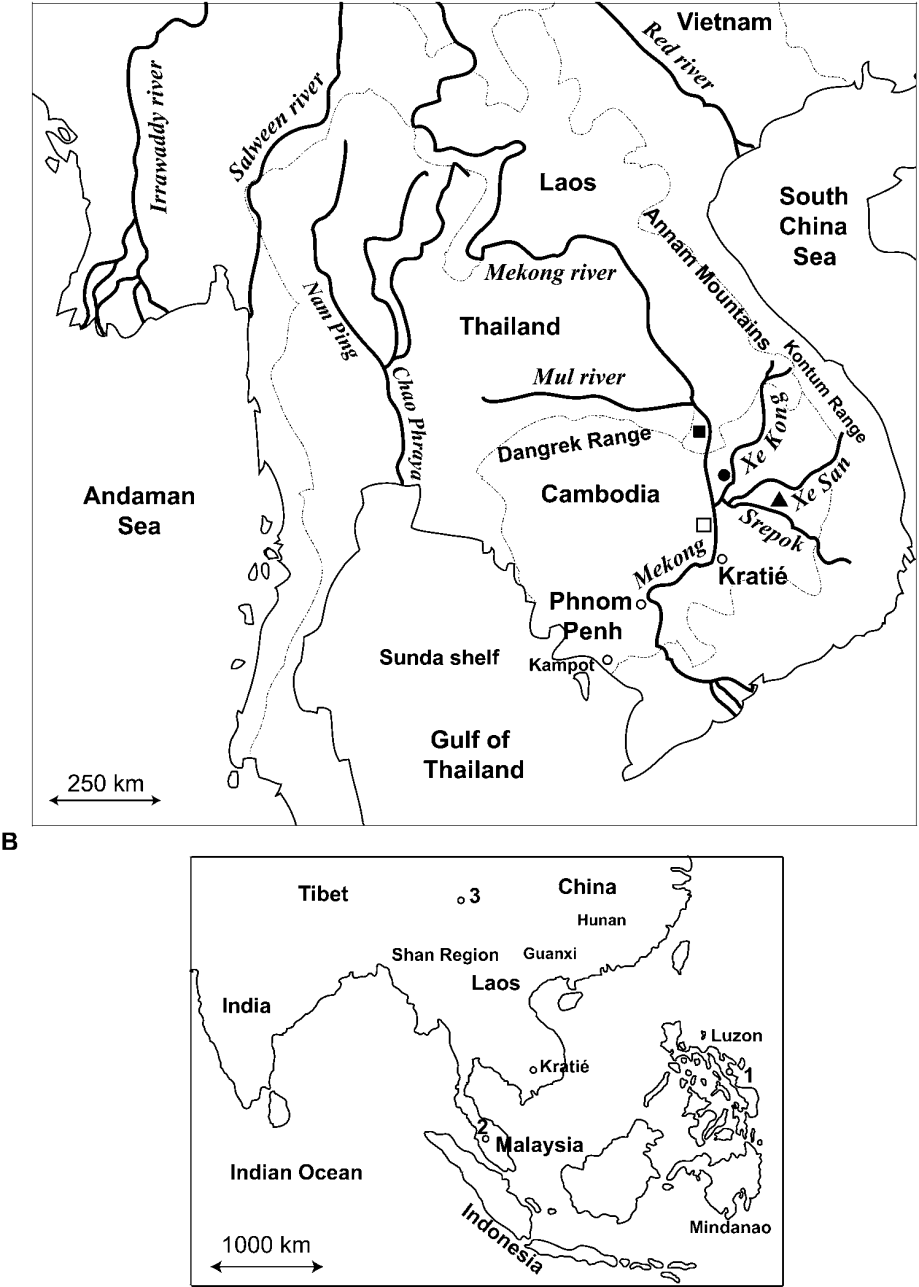
Geographical distribution of sampling sites. A The lower Mekong Basin (Central Sundaland), showing the major rivers draining the region and those sampled during the study. The approximate locations of Khong Island (▪) HXK, Sa Dao (•) SDO, Lumphat (▴) LMP, and San Dan (□) SDN are indicated. The bold lines indicate river courses, whereas the broken lines show the approximate international boundaries. B Eastern Asia showing the localities at which samples were taken. 1. Sorsogon, Luzon, Philippines, 2. Baling, Pahang, West Malaysia, 3. Mianzhu, Sichuan, China PR. Scale approximate.

Schistosomiasis in humans along the lower Mekong river (specifically Cambodia and southern Laos) was first recognized in 1957 [1] and has proven a persistent public health problem in the region [2]. The species involved is the parasitic blood fluke *Schistosoma mekongi* Voge, Buckner & Bruce 1978, which uses the caenogastropod snail *Neotricula aperta* (Temcharoen, 1971) (Gastropoda: Pomatiopsidae: Triculinae) as intermediate host. Published records identify the following foci of *S. mekongi* transmission: Ban Hat-Xai-Khoun, Khong Island, southern Laos [3] ; Kratié in Kratié Province, northeastern Cambodia, approximately 180 km downstream of Khong Island [4]; and San Dan, Sambour District, also in Kratié Province [5] (Fig. 1A). Prior to 1994 up to 40% of the admissions to Kratié hospital were schistosomiasis-related and deaths were common place [5]. Following mass treatment with the anthelmintic Praziquantel, the prevalence in school children in Kratié Province fell from 40% in 1994 to 14% in 1995 [6]. In Laos, at Khong Island, a nine year Praziquantel intervention programme reduced the prevalence among village children from 51% to 27% [2].

There has been recent optimism regarding the possible complete control of *S. mekongi* infection [7],[8]; however, this may be unfounded, not only because of the persistence of infection in reservoir hosts [9],[10],[11], but also because the range of *N. aperta* (and therefore the potential range of the disease) has been underestimated. No new cases of severe morbidity have been reported in Cambodia since 2002, but three new cases of human infection were reported in 2005 [12]; this highlights the resilience of transmission in the face of seven years of mass chemotherapy (beginning 1996). To date all published molecular studies on *S. mekongi* transmission, and much of the control efforts, have been restricted to little more than a 200 km stretch of the lower Mekong river. Recent surveillance of tributaries of the Mekong river, that drain the Annam highlands (Fig. 1A), and of other river valley systems, have revealed new *N. aperta* populations. Recently 11 new *N. aperta* populations, involving six new river systems in Cambodia and Laos, were reported [13]. Many of the new populations lay outside the Mekong river valley, most were reported from the upper Xe Kong river valley. Prior to these studies the total population at risk was estimated to be 120,000 people [2]; however, after taking into account these new populations in areas beyond the lower Mekong river, the potential affected population rises to over 1.5 million. The findings also suggested that areas cleared of *N. aperta* by control efforts in the Khong Island area or in Kratié could be rapidly recolonized by snails from inaccessible populations in the tributaries draining the Annam mountains. In 2004 *S. mekongi* was detected in an *N. aperta* population at Sa Dao in the Xe Kong river of Cambodia (Fig. 1A) [13]; this was the first published direct evidence for the ongoing transmission of *S. mekongi* outside the Mekong river and further suggests that control of Mekong schistosomiasis will be problematic.

Phylogeographies (incorporating data on DNA-sequence variation) have been used to study the evolutionary radiation of Asian *Schistosoma* Weinland, 1858 [14]–[16], their relationships with other *Schistosoma* [17], and their snail intermediate hosts [18]–[20]. Earlier studies suggested that *Neotricula* arrived in Laos after dispersing southwest from Hunan (China) via the Red river (Fig. 1A) [20]; this at a time when the Yangtze and Red rivers shared a common course some 400 km further West than at present (prior to Pleistocene tectonic events affecting this region [21]). Davis (1992) [18] used snail phylogenies, and Attwood et al. (2002) [16] used DNA-sequence data for *Schistosoma*, to argue that *Schistosoma japonicum* Katsurada, 1904 and subsequently *S. mekongi* diverged from an antecedent resembling *Schistosoma sinensium* Bao, 1958 in the Shan region of China and Myanmar (Fig. 1B). In this case *S. mekongi* would be expected to occur in northern and central Laos and even in Vietnam.

Recent DNA-sequence based phylogenies show *Schistosoma malayensis* Greer et al., 1988 as a sibling species of *S. mekongi* [22]–[24]. Indeed, the two species differ only in terms of intermediate host, life cycle parameters (e.g. length of pre-patent period), and biogeography [25]. Examination of DNA-sequence variation between the intermediate hosts of *S. malayensis* and *S. mekongi* estimated their divergence at 5 Ma (million years ago) [26]; however, palaeogeographical models suggested that the two species were separated less than 1.5 Ma [26]. Després et al. [27] used an ITS2 molecular clock rate of 0.3–0.8% per Myr to date the divergence of *S. japonicum* from African species at 24–70 Ma. In contrast, Attwood et al. [28] estimated a divergence date of only 12 Ma on the basis of palaeogeography, available dispersal tracts and the radiation of definitive host groups. Després et al. [27] suggested that the divergence of *S. haematobium* in Africa, from other species infecting animals, was triggered by the colonization of savanna areas by hominids (1–10 Ma). Similarly, the wide host range of *S. japonicum* (which is a true zoonosis) has been explained as a result of very recent transfers from animals to modern humans [29].

In the present study samples of *S. mekongi* were taken from all published foci of infection and from a previously unknown population in Lumphat District of Northeast Cambodia; these enabled the first intraspecific study of *S. mekongi*. Surveys were performed in Lumphat because the region was accessible and there have been suggestions of past transmission in Rattanakiri Province [30]. The Lumphat taxon showed morphological differences (larger eggs and cercariae) from other populations. The work was undertaken to confirm the status of the Lumphat and Sa Dao taxa as *S. mekongi*, to provide the first divergence date estimates for the radiation of *S. mekongi* in Southeast Asia (that can be used as priors in future studies), and to estimate a phylogeny for Southeast Asian *Schistosoma* which can be compared with phylogenies and historical biogeographical hypotheses for the intermediate hosts. The public health implications of the reported data are also considered.

## Methods

### Sampling

Samples were taken in Cambodia, Laos and Pahang State, West Malaysia. Table 1 gives details of sampling sites, laboratory lines, dates of collection, sample codes, whilst Table 2 details other sources of DNA sequence data. Adult worms were obtained following published methods [16] using the hamster (*Mesocricetus auratus*), as the laboratory definitive host, and cercariae from naturally infected snail intermediate hosts, but with the following exceptions. The MAL sample was obtained from field trapped rodents by perfusion [31] and the JAP sample was obtained from laboratory lines. Tegumental features (tubercles, spines, etc.), gross internal anatomy and egg morphology were used to identify the worms. DNA was preferentially extracted from females or from separated worm pairs for which eggs had been observed and identified *in corpo*. Species identification followed relevant publications for *S. japonicum* [32]–[34] and for *S. mekongi* [32],[33],[35].

**Table 1:** Taxa sampled, collecting sites (populations) and dates of field collection for samples used during the present study.

Schistosoma species	Collecting site	Date (dd/mm/yy)	Coordinates	Isolate details	Population code	River
*S. japonicum*	Luzon, Philippines	29/01/04	12°59′20″N; 125°00′05″E	L(1989)	JAP	-
*S. malayensis*	Baling, West Malaysia	21/08/03	05°42′30″N; 100°57′30″E	N (2)	MAL	Perak
*S. mekongi*	Ban Hat-Xai-Khoun, Laos	24/04/00	14°06′30″N; 105°51′45″E	M (4)	HXK	Mekong
*S. mekongi*	Sa Dao, Stung-Treng, Cambodia	25/04/03	13°36′45″N; 106°06′00″E	M (3)	SDO	Xe Kong
*S. mekongi*	San Dan, Kratié, Cambodia	28/04/03	12°44′30″N; 105°59′30″E	M (3)	SDN	Mekong
*S. mekongi*	Lumphat, Rattanakiri, Cambodia	26/04/04	13°29′30″N; 106°51′00″E	M (4)	LMP	Srepok

Life cycle stage sampled: L, from a laboratory line (with year line established); M, sampled from a new infection using cercariae from naturally infected snails; N, natural infection, i.e. sampled from a natural infection in field trapped definitive hosts. Numbers of infected snails or field trapped rodents (as applicable) used are given in parentheses under ‘Isolate details’.

**Table 2:** DNA sequence data obtained from published sources and used in the present study.

Taxon	Population	Locus	GenBank accession number	Publication	Population code
*S. incognitum*	Phitsanulok, Thailand	COI	AY157201	Lockyer et al. [22]	PHS
*S. incognitum*	Phitsanulok, Thailand	12S	AF465915	Attwood et al. [16]	PHS
*S. sinensium*	Mianzhu, China PR	COI	AY157197	Lockyer et al. [22]	SIN

### DNA amplification and sequencing

DNA was extracted from single adult worms using a standard method [36]. Sequence variation was assessed at two loci, being partial sequences of the mitochondrial (mt) cytochrome oxidase subunit I gene (*cox1*) and the small ribosomal-RNA gene (*rrn*S), here denoted as COI and 12S loci respectively. Sequences of the oligonucleotide primers used in the PCR for the amplification of *rrn*S locus are published elsewhere [16]. The *rrn*S region amplified corresponded approximately to positions 11433–11760 in the complete mt genome sequence of *Schistosoma spindale* Montgomery, 1906 (see Littlewood et al. [37]). The COI locus was amplified using the HCO-2198 and LCO-1490 primer pair [38]; the region amplified using this primer pair corresponded approximately to positions 10224–10851 on the same complete mt sequence. Further details of the data set (including sample sizes and GenBank accession numbers) are given in Table 3. The efficiency of the PCR varied considerably between populations and, in some cases, this effect and the small number of worms available to us, led to a low number of replicates for some populations.

**Table 3:** Loci sequenced and sample sizes for the taxa collected during this study.

Schistosoma species	Code	Loci sequenced		GenBank Accession number
		(No. worms sampled/length of aligned sequence)		COI/12S
*S. japonicum*	JAP	COI (1/621)	12S (4/324)	EF635954/EF635948
*S. malayensis*	MAL	COI (4/621)	**12S** (8/323)	EF635956/EF635950
*S. mekongi*	HXK	COI (6/621)	12S (6/323)	EF635953/EF635947
*S. mekongi*	**SDN**	**COI** (1/621)	**12S** (8/323)	EF641269/EF641270
*S. mekongi*	**SDO**	**COI** (4/621)	**12S** (4/323)	EF635957/EF635951
*S. mekongi*	**LMP**	**COI** (2/621)	**12S** (8/323)	EF635955/EF635949
*S. sinensium*	SIN	-	12S (4/325)	-/EF635952

Loci for which no corresponding sequence data had been previously published for the taxon concerned are emboldened. Populations (Code) previously unknown in phylogentic studies are also shown in bold. Sequence lengths are given in base pairs. For explanation of codes see Table 1.

Two mt genes were selected because, with their maternal pattern of inheritance, and smaller effective population size, they were considered to represent potentially better recorders of phylogenetic events at the intra-specific to sibling species level. In addition, the loci targeted were those within regions previously shown to exhibit ideal levels of variation in *Schistosoma* for this type of study [16], and those which had been used in earlier studies so that data were already available for the outgroup and for comparisons with related taxa.

Total genomic DNA was used as a template for PCR amplification on a Progene thermal cycler (MWG) employing standard PCR conditions [39]. Unincorporated primers and nucleotides were removed from PCR products using the QIAQuick PCR purification kit (QIAGEN). Sequences were determined bidirectionally, directly from the products by thermal-cycle-sequencing using Big Dye fluorescent dye terminators and an ABI 377 automated sequencer (Perkin-Elmer), following procedures recommended by the manufacturers. DNA extracts were not pooled and one DNA sequence thus represented one worm. Sequences were assembled and aligned using Sequencher (version 3.1 Gene Codes Corp. Ann Arbor, Michigan). DNA sequences for both strands were aligned and compared to verify accuracy. Controls without DNA template were included in all PCR runs to exclude any cross-over contamination.

### Choice of substitution model and preparation of data

Consensus sequences for the populations sampled were grouped together into sets of aligned sequences of equal length (one set for each locus), such that all taxa were represented in each set (Table 3). In addition, the COI and 12S sequences for each population were concatenated and aligned to form a combined data set. No intrapopulation variation was found among the sequences. Outgroup sequences were taken from the GenBank for *Schistosoma incognitum* Chandler, 1926 from Central Thailand. Phylogenetic analysis was conducted using both a solely maximum likelihood (ML) approach and a Bayesian method (BM). The present data showed significant variation in the rate of substitution among sites, together with considerable bias among the six different types of nucleotide substitutions. In such cases, ML-based methods are considered more robust than most other commonly used phylogenetic methods, as they permit a better optimized model of substitution [40]. The three data sets were analysed separately by ML and BM.

A suitable substitution model was selected using an hierarchical test of alternative models as implemented in Modeltest v. 3.06 [41]. A General Time Reversible model, with estimates for among site rate heterogeneity (GTR+G), was the model selected for the COI data (the-ln likelihood for this model was 2038.1306, whereas the–ln likelihood for the next more complex model was 2037.0510; X^2^ = 2.1592, *P* = 0.0709). The Hasegawa, Kishino and Yano model, again with estimates for among site rate heterogeneity (HKY+G), was the model selected for the 12S data (the-ln likelihood for this model was 850.6237, whereas the–ln likelihood for the next more complex model was 850.1796; X^2^ = 0.8882, *P* = 0.1730). The data were partitioned and the appropriate model applied to each partition during the analyses. The data were tested for substitution saturation using plots of the numbers of transitions and transversions against the ML genetic distance (following DeSalle et al. [42]). The indications of these plots were further evaluated using the entropy-based test [43] as found in the DAMBE (v. 4.5.29) software package [44], which provides a statistical test for saturation. Statistics relating to polymorphism (see Table 4) were computed using DNAsp (v. 3.51) [45]. The incongruence length-difference (ILD) test [46], as implemented in PAUP* (v. 4.0b10; [47]), was used to test for homogeneity between the COI and 12S data partitions prior to combining them; the test was applied to informative sites only [48]. In all analyses, gaps were treated as missing data and all characters were run unordered and equally weighted.

**Table 4:** Statistics relating to each data set used in the analyses.

	COI	12S	COI+12S
Length	623	331	954
Taxa	8	8	7
Length (no gaps)	619	315	933
H	0.893±0.111	0.857±0.117	1.000±0.076
D	0.147±0.032	0.0881±0.029	0.140±0.030
PS	214 (76)	75 (17)	289 (93)
PT	>0.10	>0.10	>0.10

Length, total number of sites in alignment; Taxa, number of taxa; Length (no gaps), total number of sites excluding those with alignment gaps; H, haplotype diversity; D, Jukes-Cantor corrected nucleotide diversity based on the total number of mutations; PS, number of polymorphic sites (with parsimony informative sites in parentheses); PT, significance of Tajima's test for neutrality based on the total number of mutations. The COI+12S dataset included only the unique haplotypes.

### Phylogeny reconstruction: starting parameter values and priors

For the ML method heuristic searches were performed (under the respective model and starting parameters indicated by Modeltest) using PAUP* with random addition of sequences (10 replicates) and tree-bisection-reconnection branch-swapping options in effect. Nodal support was assessed by bootstrap with 5000 replicates. Starting parameters for the BM were taken from Modeltest; these were then “optimized” using a ML method with the Brent Powell algorithm in the phylogenetics software suite P4 [49]. The values from these optimizations were used as starting parameters for the first Bayesian analyses. A Metropolis-coupled Markov chain Monte Carlo sampling process (McMcMC) [50] was used to search the parameter space of our evolutionary model and compute the posterior probability density.

Although a direct ML method was used in this study this was mainly to afford comparisons with earlier work. The final inferences were made using a BM; this is in accordance with a growing opinion that Bayesian phylogenetic analysis is not only faster in terms of computing time (for analyses with an equivalent level of confidence) but also statistically superior to a solely ML method [51]. For example, such methods do not assume approximate normality or large sample sizes as would general ML methods [52]; they also allow the incorporation of prior information about the phylogenetic process into the analysis. In this study P4 was used to apply the BM; this employs the same method as MrBayes [53] but allows consideration of unresolved trees (i.e. polytomies) and provides an automated (iterative) procedure for tuning the McMC acceptance rates to acceptable levels. The McMC was thereby tuned to give proposal acceptance rates between 10 and 70% for each data partition (this required over 5,000 replicates). The P4 analyses (except for those using the polytomy prior) were repeated in MrBayes (3.1.2) to reveal any topological disagreement.

The priors specified for the BM generally followed the default values found in MrBayes; a flat Dirichlet distribution was set as the prior for the state frequency and for the rate set priors (*e.g*., revmat, tratio), the branch lengths were unconstrained. A polytomy proposal was set as either zero (*i.e*., no favouring of multifurcations) or as *e, e^2^* or 10 to examine the effect this has on the posterior probabilities of the clades found; this implements a move (proposed by Lewis et al., 2005) to counter the problem of the spuriously high posterior clade probabilities returned by MrBayes relative to corresponding ML analyses [54]. During the Bayesian analysis, model parameters and relative rates were set to be freely variable; there were four discrete rate categories for the Γ-distribution.

Convergence of the McMC was assessed by plotting split support (for the *S. malayensis/mekongi* partition) for consensus trees over different generation time windows; the generation of convergence was considered to be that at which the support reached a plateau. In this way, a burnin of 400,000 generations was found to be adequate for all the analyses in this study. Posterior probabilities were then estimated over 900,000 generations beyond the assumed point of stationarity. Four simultaneous Markov chains were run (one cold, three heated) and trees were sampled every 10 generations, two such runs were performed simultaneously. After 900,000 generations (post-stationarity) the average standard deviation of the split frequencies (between the two runs) was checked; the McMC was considered complete if this SD was <0.01.

### Estimation of molecular clock rates

Likelihood ratio tests (LRTs) were performed to assess the applicability of a molecular clock across the whole phylogeny [55]. The program BEAST (1.4.3) [56],[57] was used to estimate the rates. BEAST implements a Bayesian method for the simultaneous estimation of divergence times, tree topology and clock rates; this method is currently considered superior to other approaches (e.g., non-parametric methods such as NPRS [58] or penalized likelihood methods [59], particularly for phylogenies with a low time depth, because it can allow for uncertainty in dates assigned to calibration points and does not require untested assumptions about the pattern of clock rate variation among lineages [60]. The procedure involves the user specifying both a phylogenetic model (a model of evolutionary history; the tree model) and a clock model (of substitution and rate variation); however, the likelihood calculation is based on the clock model only. Rate variation between adjacent branches is assumed to be uncorrelated, as these rates did not show autocorrelation in recent studies [61]. BEAST can implement several combinations of tree and clock models, but for several combinations it was not possible to obtain a stable result (between replicate McMC chains) or a sufficient effective sample size (ESS) for parameter estimates (sufficient being >200). The program TRACER (1.3) [62] was used to check convergence of the chains to the stationary distribution by visual inspection of plotted posterior estimates and to summarize parameter estimates, errors and confidence intervals. For those models which gave stable results, the ratio of the marginal likelihoods (with respect to the prior) of alternative models (i.e., the Bayes Factor) was used to choose between them [63] (who used importance sampling and the harmonic mean of the sampled likelihoods as an estimator); this does not maximize the likelihoods but averages them over the parameters involved. The calculation was implemented using BEAST (1.5 alpha) following [64]. Divergence dates (Table 5) were taken from the Bayesian posterior distribution of the divergence of the taxa concerned.

**Table 5:** Results of a Bayesian estimation of divergence times (in millions of years) for nodes representing the most recent common ancestor (MRCA) of relevant clades.

Parameter	Mean±S.D.	ESS	95% HPD
			Lower/Upper
Likelihood	−2864.183±0.079	4734	−2871.342/−2856.524
TMRCA (ingroup)	4.608±0.010	15016	2.956/6.202
TMRCA (*japonicum*)	3.855±0.023	1140	1.156/6.081
TMRCA (*malayensis*)	2.452±0.095	520	0.195/5.177
TMRCA (*mekongi*)	1.318±0.055	1184	0.025/4.021

ESS, effective sample size; HPD, the 95% highest posterior density interval (equivalent to a confidence interval); Likelihood, posterior log likelihood (of the model, given the observed data); TMRCA, time to MRCA (/Ma). Explanation of clades: ingroup, MRCA of all taxa excluding the outgroup (*S. incognitum*); *japonicum*, MRCA of *S. japonicum* and all ingroup taxa excluding *S. sinensium*; *malayensis*, MRCA of *S. malayensis* and all *S. mekongi* taxa; *mekongi*, MRCA of all *S. mekongi* taxa.

The greatest benefit of using a Bayesian method for dating is that the specification of prior distributions can be used to ensure that the analysis realistically incorporates the uncertainty associated with the calibration points used [65]. The models and the priors for the BEAST analyses were set as follows. The tree model prior assumed that divergence patterns followed a Yule process where symmetrical trees are considered more probable (i.e. a simple uniform probability of speciation); this prior and a basic coalescent model (which assumed a constant population size over the time period concerned) were used to obtain the starting tree for the analysis. The clock rates were drawn from either a log normal distribution or an exponential distribution, which were then used to specify the probability of a certain substitution rate on a particular lineage during the McMC. The GTR+G model was applied to the COI partition and HKY+G to the 12S (GTR+ss (ss, site specific rates) could not be used owing to a paucity of polymorphic sites at the first codon position, which causes the BEAST analysis to stall). A normal clock rate prior was specified (0.035±0.0071 substitutions per site per Myr); this was based on rates for *S. mansoni* and *S. incognitum* estimated elsewhere [66]). A normal prior (5.0±0.1 Ma) was applied to the TMRCA for the ingroup; this corresponded to the second major Himalayan orogeny which could have isolated central Asian taxa from those of the Orient [28]. For the final parameter estimates three independent runs of 130 million generations were combined to give a final set of 390 million states; the burnin was set to 10%.

## Results

### Sequence analysis

Table 4 provides basic statistics for the two loci and the combined data. The COI data appeared the most informative having a greater proportion of parsimony informative polymorphic sites (12.3% of the total number of aligned sites, excluding gaps, compared with 5.4% for 12S). Similarly, 34.6% of positions were polymorphic in the COI data set (of these 35.5% were informative sites, the remaining 64.5% being singletons) and only 23.8% in the 12S set (of which 22.7% were informative). For the COI data 201 mutations were inferred of which 121 (60.2%) were synonymous and 80 (39.8%) were amino acid replacements. The test of Xia et al. [43] suggested that there were no significant levels of substitution saturation at either locus (I_SS_<I_SS.C_, *P*<0.0001, a lack of statistical significance here would imply a poor phylogenetic signal). Table 4 also shows that the nucleotide diversity (D) was greater for the COI data. The haplotype diversities for the full taxa set (H, Table 4) show that not all taxa had unique haplotypes. In the COI set the HXK, SDN and SDO samples shared a common “lower Mekong river” haplotype. Among the 12S sequences SDN, SDO, LMP and *S. malayensis* shared a common haplotype; that *S. malayensis* was indistinguishable at this locus highlights its close relationship with *S. mekongi*. In the combined COI+12S data set each taxon was represented by a unique haplotype, except for SDN (which was identical in state to SDO) which was excluded from the Bayesian analysis. In all cases the test of Tajima (1989) [67] failed to refute the hypothesis of neutral evolution. LRTs for all data sets failed to support the hypothesis that the different lineages had been evolving at the same rate (-ln likelihood with a clock enforced 2932.5967, without clock 2921.57447; X^2^ = 22.04, *P* = 0.0005).

### Phylogeny reconstruction

**Figure 2:**
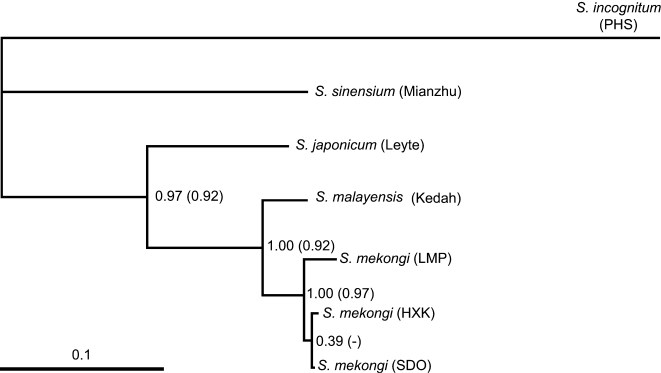
Phylogram with maximum posterior probability, for the combined COI and 12S data set and the *Schistosoma sinensium* group, from a Bayesian method (outgroup *S. indicum*). Numbers assigned to each node represent the posterior probability that the hypothesis represented by this bi-partition, and under all parameters of the model, is correct given the observed data (the numbers in parentheses are bootstrap support values for a corresponding Maximum Likelihood analysis, 5000 replicates). Posterior probabilities shown are for a basic model, allowing a polytomy proposal of *e^2^* reduces the probabilities to 0.95, 0.89, 0.64, 0.38 (respectively, moving from left to right on the tree).

Phylogenies estimated using ML showed the same topology with all three data sets, aside from differences due to the number of distinct haplotypes. An LRT comparing the GTR+G and GTR+ss models for the COI data indicated a significant difference between them (X^2^ = 8.71 *P* = 0.0128, d.f. = 2) favouring GTR+ss, consequently this model was used for the COI partition in the Bayesian analysis of the combined data set (but GTR+G was used in the BEAST analyses, see METHODS final section). Figure 2 shows the tree resulting from phylogenetic estimation using BM and the COI+12S data set; this tree is identical to that of the ML analysis except that with ML there is an unresolved trichotomy for the three *S. mekongi* populations. Performing the BM with the polytomy prior turned off resulted in posterior probabilities >0.97 (except for the HXK/SDO node at 0.39) (Fig. 2), increasing the prior to *e* led to a slight drop in the probabilities, further increases to *e* ^2^ and 10 had little further effect. The topology and split support using MrBayes was very close to that of P4 with the polytomy prior turned off. *Schistosoma malayensis* is confirmed as a sibling species of *S. mekongi* and the *S. mekongi* populations form a monophyletic unit on the tree; the statistical support for these groupings is high (1.00). The *S. mekongi* population of LMP appears as sister to a clade comprising the HXK and SDO populations, in the *S. mekongi* lineage, but this relationship is less well supported (posterior probability only 0.39).

### Estimation of divergence times

Aside from the Yule process several more complex models of past population dynamics can be implemented using BEAST (e.g. past exponential, logistic or expansive growth and the Bayesian skyline model); however none of these gave stable results (after multiple runs of several 100 million states with tuning and prior-adjustments, or varying starting trees) or they had very low likelihoods. LRT indicated that a strict molecular clock model was inappropriate for these data (*P* = 0.0005). Consequently the Yule model was used in the final analyses in this study and the log normal and exponential clocks were compared. Table 5 shows the results of a Bayesian estimate using a Yule tree model and an uncorrelated log normal relaxed clock. Comparison of the posterior log likelihood of this model with that for the next best model (Yule process with an uncorrelated exponential clock) gave a Bayes factor of 22.35 which strongly favoured the log normal model. The TMRCA values given in Table 5 are summarized from the Bayesian posterior distribution of the divergence times of the taxa involved in the partition. The exponential clock model gave much lower TMRCAs than the statistically “preferable” log normal model; for example, TMRCA (*mekongi*) 9,760 years before present (YBP), TMRCA (*malayensis*) 45,512 YBP, TMRCA (*japonicum*) 242,690 YBP. Plots for the posterior distribution of estimates of mutation rate and the TMRCAs in the Yule/log normal analysis were bell-shaped and showed no cut-off at the upper or lower bounds; this suggested that the priors used were not restricting the range of values implied by the data [68] (this restriction was found with other model/prior combinations). The wide range of the HPDs in for the divergence time estimates in Table 5 reflects the uncertainty inherent in all molecular date estimates; this is not unusual and is a realistic feature of this method of analysis.

## Discussion

### Divergence dates

The *Schistosoma indicum* to ingroup divergence date of around 4.6 Ma (see Table 5) implies a 2.5% (of sites varying per Ma) clock for COI and a 2.0% clock for the 12S locus; these figures appear to be moderate values and compare well with published rates of 1–2% for African and South American *Schistosoma* at mt loci [27], of 3% for *S. indicum*-group taxa [28], and of 1% averaged across metazoan groups in general [69]. Attwood et al. [26] suggested a divergence date of 5 Ma for the intermediate hosts of *S. mekongi*/*malayensis*, whereas the TMRCA corresponding to this divergence for the parasites themselves in the present study was approximately 2.5 Ma. The S.D. of this estimate is only 4% but the confidence interval is wide, from *c.a.*, 200 KYBP (thousand years before present) to 5 Ma; these wide 95% confidence intervals are common for Bayesian date estimates, they are wider than those of ML based point estimates but this is only because other methods fail to account fully for the uncertainty in the estimation procedure. Attwood et al. [26] used a simple point estimate of divergence times based on pairwise genetic distances (following [70]) and relied on a general invertebrate clock for calibration. Such methodological differences may explain the incongruence between snail and parasite phylogeographies.

### Phylogeography

The phylogeny in Figure 2 shows all of the *Schistosoma mekongi* populations, including that of LMP, as lying within a monophyletic clade and this hypothesis is well supported (posterior probability = 1.00). Consequently, it appears that the *Schistosoma* found in the Srepok river is indeed *S. mekongi*; this finding has implications for schistosomiasis surveillance in Vietnam. The Srepok river originates in Vietnam and flows westwards into Cambodia. Initial studies suggested that *Neotricula aperta* evolved in northern Laos/Thailand from a lineage dispersing from India, via Tibet and Yunnan (China), along the Miocene extended upper Irrawaddy and Mekong rivers; the same historical biogeography was assumed for *S. mekongi* diverging from *S. japonicum* [71]. However, more recent work suggested an origin for both proto-*S. mekongi* and proto-*N. aperta* in Hunan or Guangxi Provinces, China, with a Yangtze-Red river radiation into Cambodia via Vietnam [2]. At least five species of *Neotricula* Davis, 1986 are known from Hunan but only one from Laos and none from Yunnan; therefore it is more likely that *Neotricula* and an antecedent of *S. mekongi* arrived in Vietnam and Cambodia directly from Hunan and not from Yunnan, via Thailand and Laos [20]. Palaeogeographical evidence appears to favour the Vietnam-Cambodia dispersal hypothesis. Much of the Annam mountain chain (which today forms a barrier between Hunan and northern Laos and Vietnam) is Mesozoic and at 1.3 Ma the only trans-Annam dispersal corridor would be the 900 km long valley of the Red river fault, which in the past ran up to 400 km closer to Laos than today [21]. The Pliocene Yangtze is also reported to have flowed along a common course with the Red river [72]. The present data yielded an estimated date for the radiation of *S. mekongi* in Cambodia of around 1 Ma; this is just before the uplift of (volcanic) highlands in Southeast Cambodia when it would have been possible for *S. mekongi* to enter Cambodia from Vietnam, just South of the Kontum range. The Srepok river population (LMP) in southern Cambodia is seen as a sister taxon to the other (Xe Kong and lower Mekong river) populations in Figure 2 and may have been early divergent. A phylogenetically basal Srepok river population would be in agreement with the idea of an *S. mekongi* radiation beginning in Southeast Cambodia; however, the support for this clade was low (posterior probability = 0.39) and only three endemic geographical regions are available for comparison.

The North to South tract, from Yunnan to northern Thailand/Laos and then Cambodia, as proposed in an earlier publication [71] cannot readily explain the absence of *S. mekongi* from suitable transmission habitats in central Laos. The only known foci of transmission are on the border with Cambodia, around Khong Island at the southern tip of Laos. In contrast, a South to North dispersal together with the Pleistocene (i.e., relatively recent) divergence date estimated here, explains the current range of *S. mekongi* as a consequence of the limited time available for dispersal from Cambodia into Laos. The Dangrek escarpment lies immediately East of HXK (Fig. 1A); these Mesozoic hills are a likely effective biogeographical barrier between Cambodia and Laos.

*S. malayensis* has been regarded as a geographical isolate derived from the *S. mekongi* radiation in Cambodia [2]; however, Figure 2 shows *S. malayensis* as sister to the *S. mekongi* clade and the divergence dates of 2.5 Ma estimated for *S. malayensis/mekongi* and around 3.8 Ma for *S. japonicum*/Southeast Asian *Schistosoma* suggest that *S. malayensis* is basal in the true phylogeny rather than a derivative of *S. mekongi*. The ancestral definitive hosts of Asian *Schistosoma* were probably rodents [20]. *S. malayensis* appears to have retained this ancestral condition, with *S. mekongi* showing derived character states, that is the ability to utilise humans and *Neotricula aperta* as definitive and intermediate hosts, respectively. *N. aperta* is a snail of larger faster rivers than the springs and primary streams to which *S. sinensium* and all other *Neotricula spp.* are restricted. The Pliocene Dong-Ngai-Mekong river could have introduced an *S. malayensis/mekongi* antecedent to the whole Sundaland drainage, with later range contraction, fragmentation and divergence. The divergence time of 2.5 Ma coincides with a major intensification of monsoon winds affecting rainfall and flow patterns in the rivers of the region [73]; this would have impacted on the distribution of the intermediate hosts and could have isolated Cambodian proto-*S. mekongi* from Malaysian *S. malayensis*.

The mean date estimates obtained here agree well with palaeogeographical data and hypotheses based on snail phylogenies. For example, the radiation of *S. mekongi* in Cambodia (dated at 1.3 Ma) correlates well with Pleistocene tectonic upheavals in the region. The severity of late Cenozoic tectonic events in Sundaland strongly suggests that the lower Mekong river (in the area of SDN and Kratié) did not occupy its present course until 5–6 KYBP [74]. Consequently, all known extant *S. mekongi* populations must have been established mid- to late Pleistocene. The Pleistocene Mekong river itself flowed further west, along the Dangrek escarpment then southwards along the Tonlé Sap of today, and across the Sunda shelf from Kampot (Cambodia) to the present day West Malaysia [75] (Fig. 1A). The divergence of the *S. sinensium* group from Central Asian lineages (here represented by *S. incognitum*) dated at 4.6 Ma agrees with the published hypothesis [20], based on snail phylogenies, that the divergence of the *S. sinensium* group was triggered by isolating events linked to the second major Himalayan uplift (5 Ma). Consequently, the date estimates obtained here are useful priors upon which further studies based on independent data may be undertaken.

### Conclusions

The work has demonstrated that transmission at all of the known foci of human schistosomiasis in the lower Mekong Basin involves *S. mekongi*, including the apparent zoonotic focus in the Srepok river. The phylogeny and divergence dates estimated, although not conclusive, correlate well with the idea of a Vietnam to Cambodia entry of *S. mekongi* into the lower Mekong region, with a subsequent South to North radiation from Cambodia into Laos. The study also demonstrates the transmission of *S. mekongi* in the Srepok river close to Vietnam. Such observations and inferences have certain public health implications. The likelihood of finding *S. mekongi* in Vietnam is increased in the light of these results. The inferred South to North dispersal of *S. mekongi* implies that it is not ecology but history which is limiting the current distribution of Mekong schistosomiasis. Further work is required into this problem, as, if we have no reason to assume that ecological conditions in Laos are unsuitable for transmission, we may expect the future spread of this disease northwards into Laos. Recent work has already demonstrated that the range of *N. aperta* is far greater than previously thought (particularly in Central Laos) [13]. The loci used here were chosen for a population phylogenetic study, with no expected intra-population variation, and not for population genetic work. Consequently, the genetic divergence among the *S. mekongi* populations was relatively small. Further work should involve additional loci and possibly also microsatellites; however, microsatellites are costly to develop and use in endemic countries and are less ideal for dating because they rely on genetic distance estimates of less certain reliability. In spite of low divergence levels, the date estimates obtained were biologically reasonable in the context of independently derived time frames and will be useful priors in future studies.
